# Synthesis of the porous Co–N-doped carbon catalysts as a durable cathode for zinc–air battery

**DOI:** 10.1038/s41598-026-40942-4

**Published:** 2026-02-28

**Authors:** Fu Niu, Jia-Ang Liu, Lin-Ting Zhao, Yi Qiao, Rui-Xia Chu, Fang-Yuan Qiu, Wan-You Huang

**Affiliations:** 1 Key Laboratory of Transportation Industry for Transport Vehicle Detection, Diagnosis and Maintenance Technology, School of Automotive Engineering, Shandong Jiaotong University, Jinan, 250357 China; 2 Shandong Motor Vehicle Exhaust Pollution Monitoring Centre, Jinan, 250000 China; 3 Yantai Dongde Industrial Co., Ltd., Yantai, 264006 China

**Keywords:** Co–N–C, ORR, Porous structure, Zinc–air battery, Chemistry, Energy science and technology, Materials science

## Abstract

Due to the exceptional ORR catalytic activity and stability, cobalt and nitrogen-doped carbon (Co–N–C) catalysts are regarded as highly promising candidates for cathode catalysts in zinc–air batteries. However, it remains a challenge to expose more stable and more efficient active sites. Therefore, this work focuses on the design and preparation of Co–N–C catalysts with a robust and porous structure. The results revealed that the robust and porous structure could be easily achieved by the template (SiO_2_)-assisted hydrothermal method. Moreover, the Co-900-50 catalyst has the highest half-wave potential, and the Co-900-100 catalyst has the highest limiting current density. Both individuals are chosen for further examination regarding their potential use in zinc–air batteries. The battery with Co-900-100 catalyst demonstrated exceptional stability across the current densities (5–20 mA cm^− 2^). Specifically, the voltage rose by 0.04 V following a 100 h discharge at a rate of 5 mA cm^− 2^, while the discharge voltage remained nearly constant at 1.24 V throughout 300 charge/discharge cycles at 5 mA cm^− 2^.

**Supplementary Information:**

The online version contains supplementary material available at 10.1038/s41598-026-40942-4.

## Introduction

Zinc–air battery is regarded as one of the best candidates for clean energy devices owing to its affordable components, safety and high specific energy density^1–3^. Nevertheless, the wide range of applications is limited by the sluggishness of the oxygen reduction reaction (ORR)^4–6^. Among the different types of catalysts, cobalt and nitrogen-doped carbon (Co–N–C) catalysts exhibit promising potential for application due to the exceptional ORR catalytic activity and stability in alkaline environments, which have been reported to be similar to that of Pt/C catalysts^7–9^.

The catalytic materials’ ORR performances are strongly linked to the specific surface area of the materials. Typically, the catalytic performance increases with a greater specific surface area^10–12^. Therefore, nanomaterials with large specific surface areas have important significance and value for the research to improve the catalytic performance. Nonetheless, nanoparticles are prone to agglomeration in the electrochemical reaction process because of their larger surface energy, ultimately causing a swift decline in both catalytic activity and stability^13^. Therefore, it is imperative to construct a unique, robust nanoporous structure to augment the durability of catalysts while preserving their catalytic efficiency for an extended duration.

Nevertheless, traditional approaches commonly employ N-rich precursors coordinated with cobalt species as the initial materials for synthesising Co−N−C single-atom catalysts (SACs)^14–19^. High-temperature pyrolysis treatment often leads to severe aggregation of cobalt species, hindering the identification of the intrinsic activity of the catalyst at the atomic level^20–22^. In addition, carbonisation at high temperatures often results in a porous structure dominated by micropores, leading to delayed mass transfer and restricted access to internal spaces, ultimately reducing catalytic activity^23^. Therefore, there is a great need for a simple and efficient method that can produce unique and robust pore structures, maximise active sites, and enhance the durability of catalysts.

The three-dimensional porous structure with abundant pores can effectively inhibit the aggregation of Co–N–C nanoparticles, while possessing a high specific surface area, allowing for the exposure of more active sites and enhancing oxygen and ion transport during the reaction process^24–27^. The three types of pore structures that can promote oxygen mass transfer in three-dimensional porous catalysts are macropores (> 50 nm), mesopores (2–50 nm), and micropores (< 2 nm). Each type of pore possesses unique functions to enhance ORR activity and durability. Large pores can store reactants, medium pores can enhance the transport efficiency of reactants, and micropores contribute to increasing the number of active centres and the total surface area of the catalyst^28^.

At present, the microspheres composed of stacked nanoparticles appear to be a favourable option, as the porous configuration aids in enhancing the movement of mass, electron transfer and the accessibility to the internal active sites^29^. The template method serves as a crucial approach for fabricating high-quality hierarchical porous carbon materials. The hard-templating approach enables precise control over the pore size and morphology of carbon materials, facilitating the synthesis of uniformly structured and well-ordered porous carbon materials^30^. Furthermore, considerable advancements have been achieved in the fabrication of porous materials by using a variety of hard templates. Of these, the hydrothermal technique stands out as a highly efficient approach for fabricating porous materials using silica templates^31–33^.

Based on the aforementioned insights, SiO_2_ was selected as the hard template. We demonstrate a facile and controllable approach for creating cobalt and nitrogen co-doped carbon catalysts with a porous structure through the hydrothermal carbonisation process in the presence of a SiO_2_ template. Upon etching the SiO_2_ template, porous carbon materials featuring a rich pore structure are acquired, including micropores, mesopores, and macropores. The rise in specific surface area reveals more active sites and creates favourable conditions for mass and ion transport. Additionally, the robust carbon shell suppresses the aggregation of Co–N–C nanoparticles, thereby ensuring outstanding catalytic stability.

The findings suggest that the presence of cobalt salt significantly influences the development of porous formations in the process of hydrothermal carbonisation of glucose. Although the optimised Co–N–C catalyst’s ORR catalytic activities are lower compared to some reports in alkaline medium, the resulting zinc–air battery shows extremely high stability at various current densities (5–20 mA cm^− 2^). Specifically, there was a minimal decrease in the discharge voltage plateaus following 300 cycles (100 h) of charge/discharge.

## Experimental section

### Synthesis

**Fig. 1:**
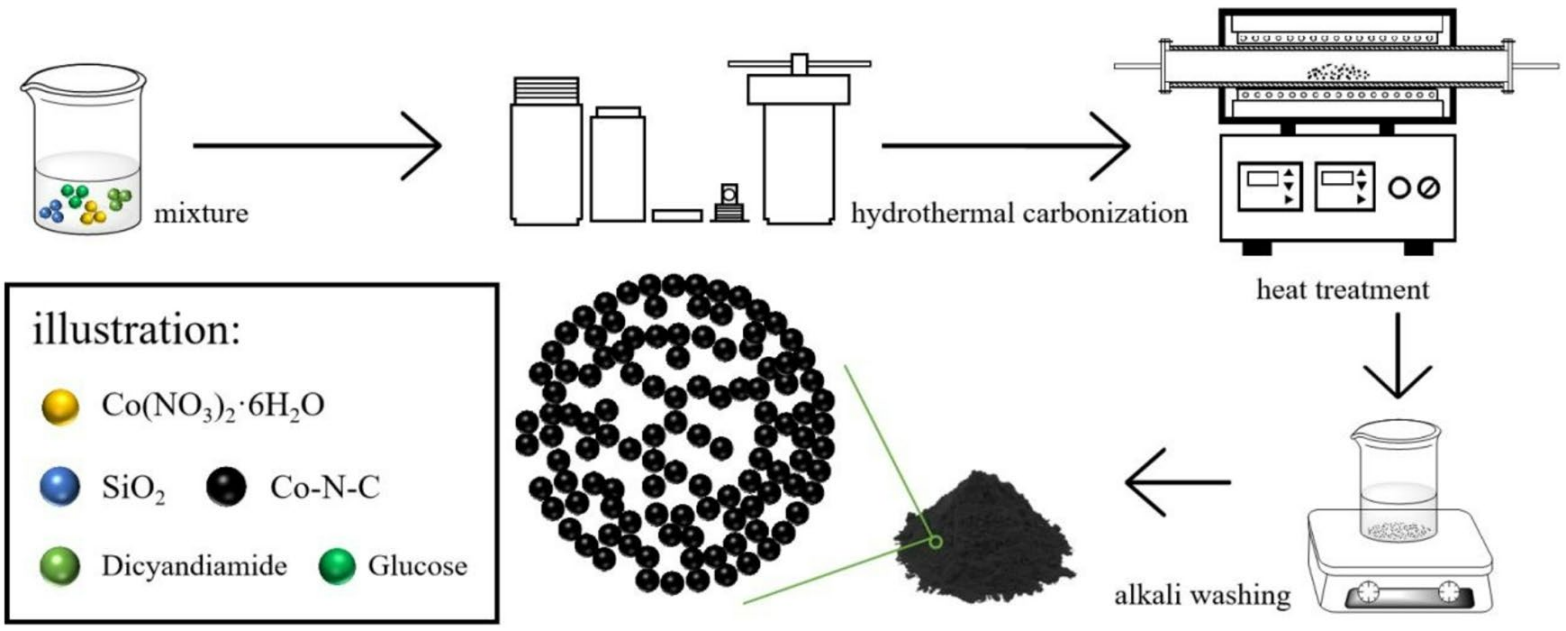
Schematic procedure for the synthesis of the catalysts.

This report only used analytical grade chemical reagents, and SiO_2_ nanospheres (the average diameter ≈ 400 nm) were fabricated via the modified Stöber method. The Co–N–C catalysts were synthesised via hydrothermal carbonisation, heat treatment, and alkali washing as shown in Fig. 1. Typically, *x* mg Co(NO_3_)_2_·6H_2_O, 30 mg SiO_2_, 20 mg glucose and 5 g dicyandiamide were dispersed in 38 ml ultrapure water under magnetic stirring for 30 min. Then, the acquired mixture was enclosed in a 100 mL Teflon-lined stainless-steel autoclave and transferred into an oven at 180 °C for 4 h. Next, brownish-black sediment was cleansed using ultrapure water and separated from the mixed solution by centrifugation. Subsequently, brownish black precipitate was subjected to heat at *y* °C for 1 h with a constant ramping rate of 5 °C min^− 1^ in N_2_ environment. To eliminate residual SiO_2_ templates, the acquired black powder was dispersed in a 2 M NaOH solution and magnetically stirred at 45 °C^34^. Following 4 h of stirring, the black powder underwent a thorough washing with ultrapure water and was harvested through centrifugation. Obtain a porous cobalt and nitrogen co-doped carbon catalyst Co-*y-x* (*x* represents the amount of cobalt salt, *x* = 30, 50, 100, 150; *y* represents the temperature of heat treatment, *y* = 700, 800, 900, 1000).

### Characterizations

The crystal structure of the samples was detected by powder X-ray diffraction (XRD) using Rigaku/Max-2550 with Cu Kα radiation. The morphology of the samples was identified using a JEOL JSM-7900 F scanning electron microscope (SEM) and a JEOL JEM-2100 F transmission electron microscope (TEM). Energy dispersive X-ray (EDX) mapping images were obtained using an EDX analyser attached to JEOL JSM-7900 F. X-ray photoelectron spectroscopy (XPS) was recorded on a Thermo ESCALAB 250Xi instrument. The micro-Raman Spectra were recorded using a Renishaw inVia Raman Microscope (532 nm excitation). Surface area was analysed via Brunauer–Emmett–Teller (BET) adsorption isotherms using a Quantachrome Nova 1200e surface area analyser.

### Electrochemical measurements

The AUTOLAB PGSTAT 302 N was used to perform electrochemical measurements in a three-electrode configuration immersed in an N_2_- or O_2_-saturated 0.1 M KOH solution. Pt wire served as the counter electrode, while an Ag/AgCl electrode served as the reference electrode. In order to fabricate the working electrode, the following steps were taken. Initially, the catalyst ink was created through the combination of 4 mg catalyst with the mixture solutions (710 µl ultrapure water, 240 µl isopropanol and 50 µl Nafion solution (5 wt%)) under ultrasonic stirring. And then, 10 µl of the catalyst ink was loaded onto a glassy carbon electrode (GCE) with an area of 0.19625 cm^2^, leading to a catalyst loading of approximately 0.2 mg cm^− 2^. The recorded potentials were normalised to the reversible hydrogen electrode (RHE) by applying the formula: *E* (RHE) = *E* (Ag/AgCl) + 0.059 pH + 0.198. The static cyclic voltammogram (CV) tests were operated at the scan rate of 10 mV s^− 1^ between 0.07 and 1.1 V (vs. RHE). Linear sweep voltammetry (LSV) was used to measure the ORR polarisation curves in the range of 1.0 to 0.1 V (vs. RHE) under the disk rotation rates at 625, 900, 1225, 1600, 2025, and 2500 rpm. All reported potentials were corrected with iR-compensation unless noted. Besides, the OER LSV curves were recorded between 1.0 and 1.9 V (vs. RHE) at a scan rate of 10 mV s^− 1^ and 1600 rpm in O_2_-saturated 0.1 M KOH electrolyte. Electrochemical impedance spectroscopy (EIS) of the catalysts was carried out at open-circuit voltage with a 5 mV amplitude and in a frequency range of 0.1–100,000 Hz.

### Zinc–air battery tests

The air electrodes were prepared as follows. Typically, the catalyst ink was acquired through the use of ultrasonic agitation in the combination ( 8 mg catalyst, 710 µl ultrapure water, 240 µl isopropanol and 50 µl Nafion solution (5 wt%)). Next, a volume of 100 µl of the catalyst ink was drop-casted onto an electrode matrix consisting of carbon cloth, a waterproof layer and a current collector of Ni foam. Approximately 1 mg cm^− 2^ was the amount of catalyst loaded. The zinc–air battery tests were performed by home-made devices. Briefly, the air electrode was used as the cathode, and the Zn plate served as the anode. Additionally 6 M KOH solution containing 0.2 M Zn(CH₃COO)₂ was used as electrolyte. The polarisation curves were obtained by utilising an AUTOLAB PGSTAT 302 N in a two-electrode configuration, and galvanostatic charge/discharge tests were conducted on a LAND battery testing system.

## Results and discussion

### Characterisation of the catalysts

**Fig. 2:**
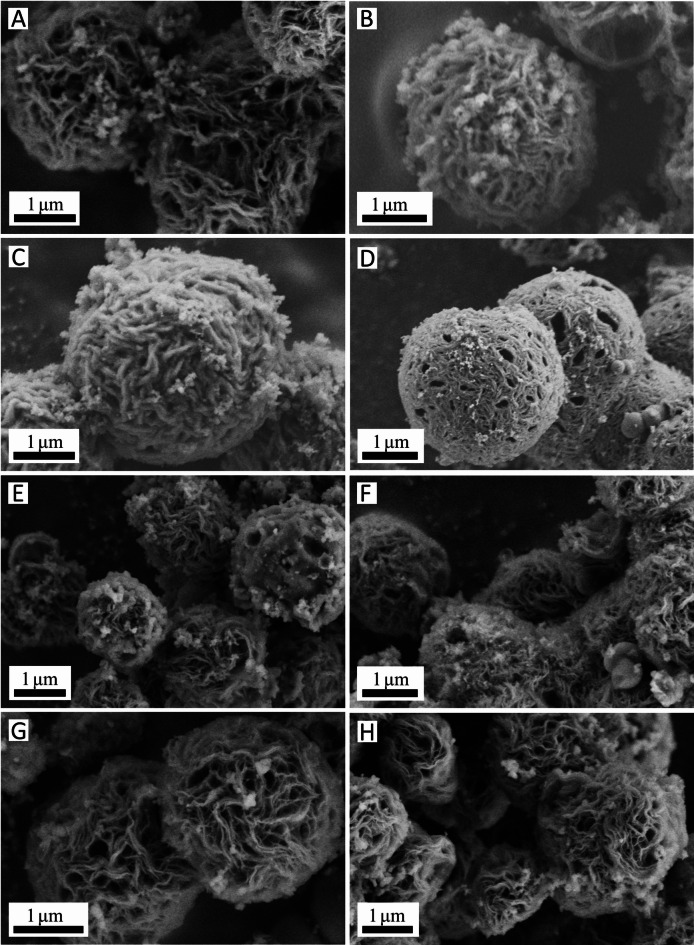
SEM images of (**A**) Co-900-30, (**B**) Co-900-50, (**C**) Co-900-100, (**D**) Co-900-150, all sample were performed at the same temperature (900 °C); SEM images of (**E**) Co-700-30, (**F**) Co-800-30, (**G**) Co-900-30, (**H**) Co-1000-30, all sample were performed at the same amount of cobalt salt (30 mg).

**Fig. 3:**
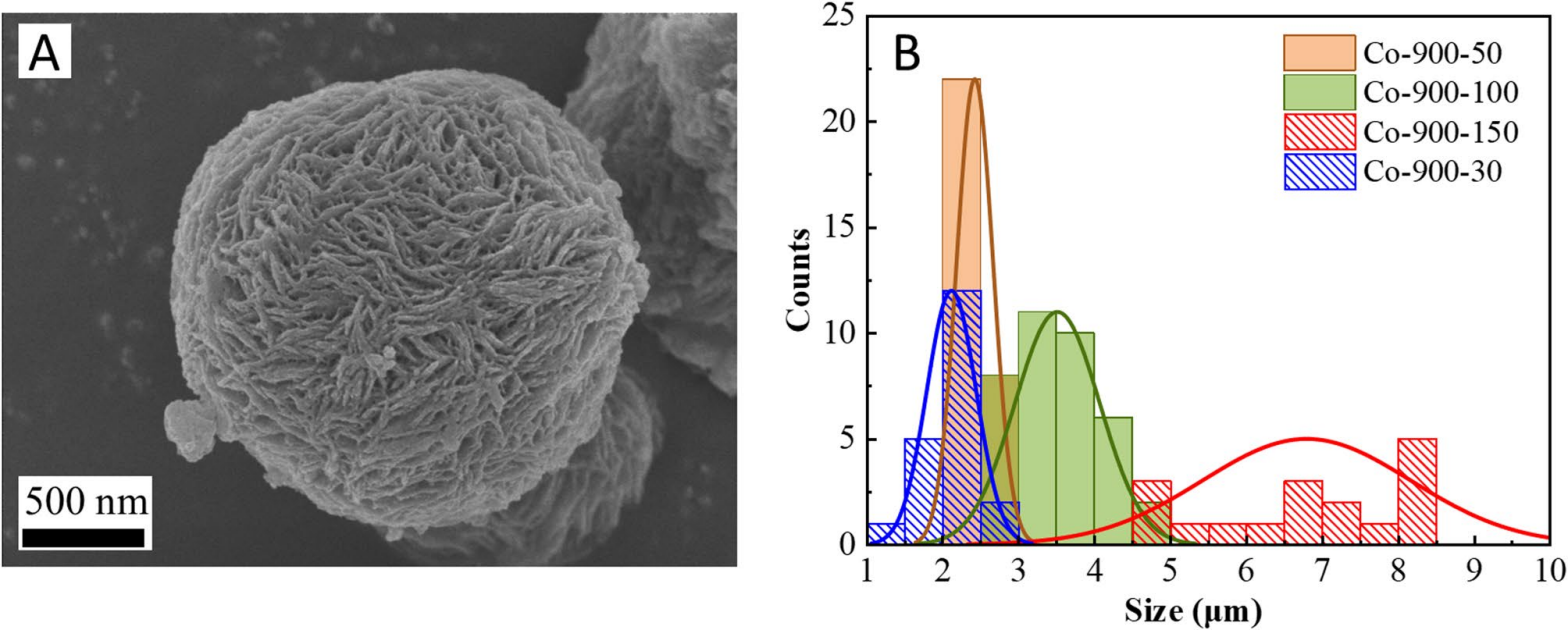
(**A**) SEM images of Co-900-50 catalyst after hydrothermal reaction, (**B**) Size distribution histogram for Co-900-30, Co-900-50, Co-900-100 and Co-900-150, showing average diameter of nanospheres to be approximately 2.1 μm, 2.5 μm, 3.5 μm and 6.7 μm, respectively.

**Fig. 4:**
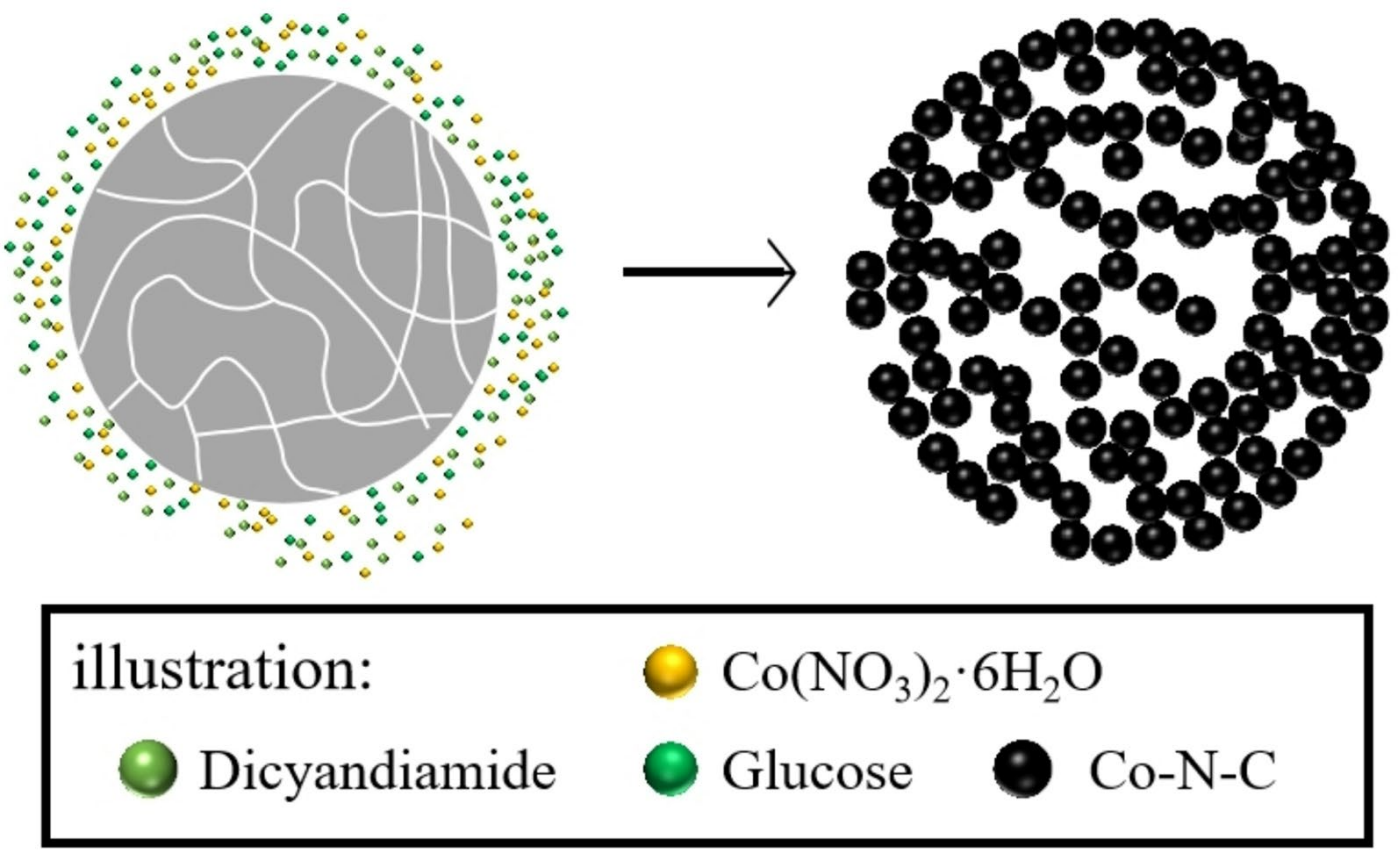
Schematic of the formation of the porous structure of the catalyst.

**Fig. 5:**
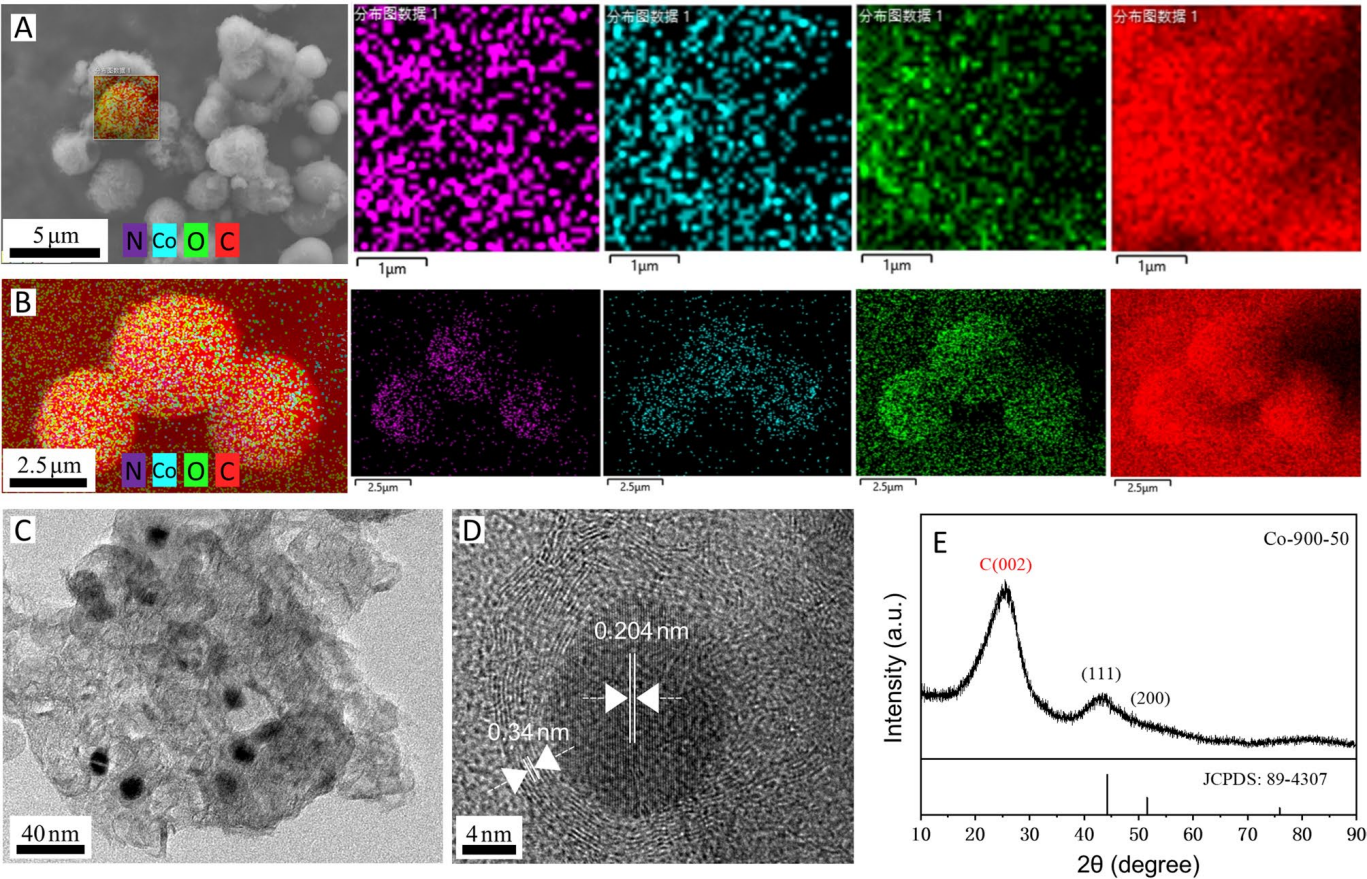
(**A**) EDX element mapping images of Co-900-50, (**B**) EDX element mapping images of Co-900-100, (**C**) TEM image, (**D**) High-magnification TEM image, (**E**) XRD pattern of Co-900-50.

**Fig. 6:**
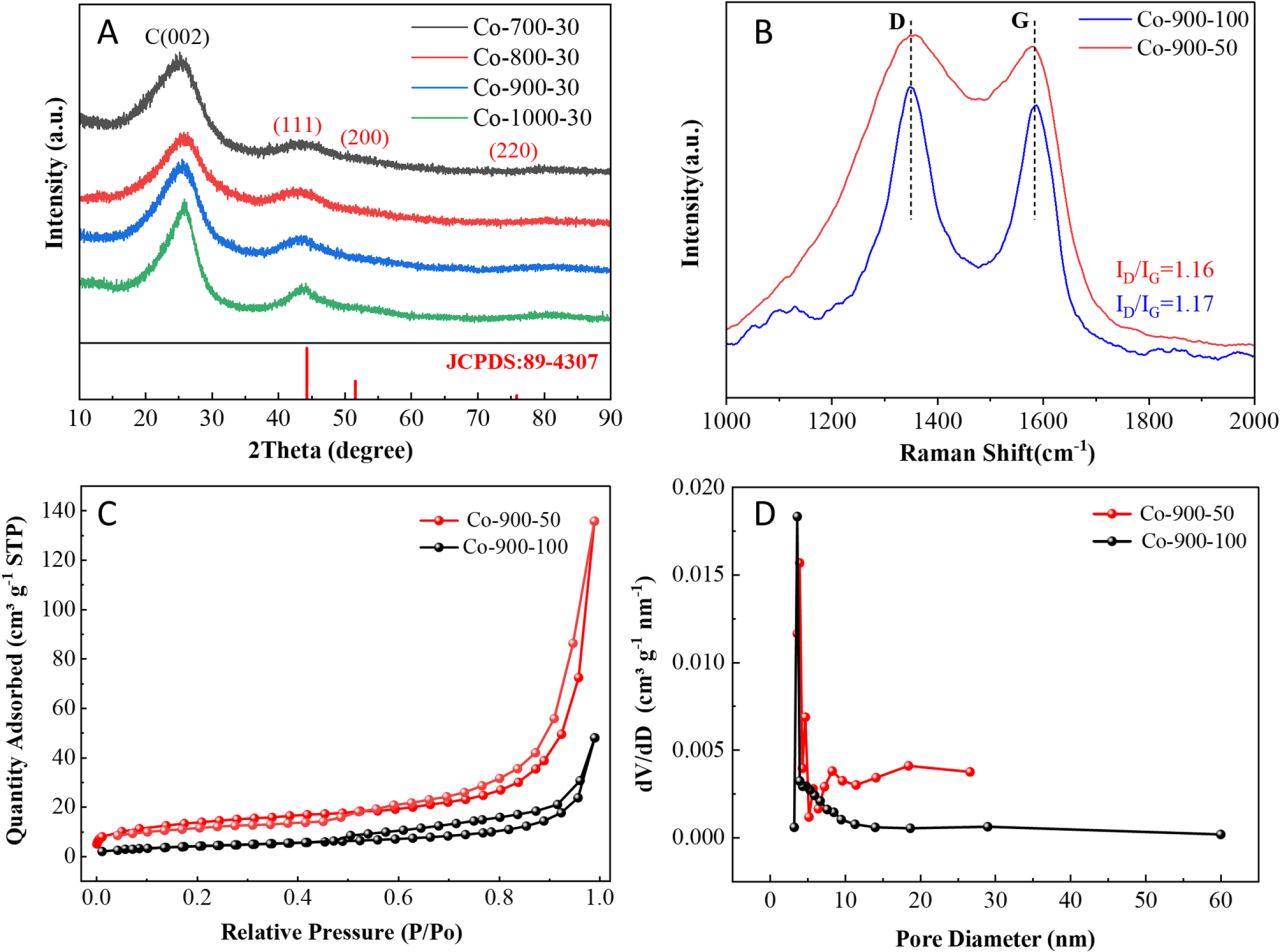
(**A**) XRD patterns at different sintering temperatures, (**B**) Raman spectra of Co-900-50, Co-900-100, (**C**) N_2_ absorption and desorption isotherms, (**D**) BHJ pore size distribution curves of Co-900-50 and Co-900-100, respectively.

Figure 2 displays the SEM images of the samples. The increase in the amount of cobalt salt at the same sintering temperature leads to a noticeable enhancement in the density and size of the microsphere (Fig. 2A,D). As shown in Fig. 3B, the average size of Co-900-30, Co-900-50, Co-900-100 and Co-900-150 was estimated as ~ 2.1 μm, ~ 2.5 μm, ~ 3.5 μm and ~ 6.7 μm, respectively. This indicates that Co-900-50 may possess a larger specific surface area compared to Co-900-100. The size distribution histogram is shown in the supporting information (Fig. 3B). In contrast, the morphology of the microsphere was little changed as the heat treatment temperature increased under the condition that the same amount of cobalt salt was utilised (Figs. 2E, 2H). The schematic of the formation of the porous structure of the catalysts is shown in Fig. 4. Co(NO_3_)_2_ 6H_2_O, glucose and dicyandiamide can be immersed into the inside of the SiO_2_ particles through the pore canal belonging to the SiO_2_ particles. Because of the strong reducing action of carbon derived from the decomposition of dicyandiamide, this can lead to larger pore canals and an increased number of pore canals, which are conducive to the improvement of catalytic performance. To verify the formation steps of the porous structure, SEM was performed on the catalyst after the hydrothermal reaction. The size of the Co-900-50 catalyst was estimated as ~ 3.1 μm (Fig. 3A), which is consistent with SEM images of the catalyst after high-temperature pyrolysis. The experimental results indicate that the porous structure is formed during the hydrothermal reaction process. Based on the above findings, the performance of the Co–N–C catalysts can be significantly adjusted by varying the quantity of cobalt salt. The cobalt mass loadings of Co-900-50 and Co-900-100 catalysts are 2.0864% and 3.5597%, respectively (Table S1). Next, Co-900-50, as a typical representative, further undergoes analysis using EDX, TEM and XRD to investigate the elemental composition and distribution, the internal structure and the crystal structure of the catalyst, as depicted in Fig. 5. Firstly, the initial findings from EDX analysis (Fig. 5A) indicate that Co-900-50 consists of carbon, cobalt, nitrogen and oxygen. Furthermore, the individual components are uniformly spread throughout Co-900-50. Similarly, the EDX images of Co-900-100 (Fig. 5B) give a distinct outline of the constituent elements, clearly revealing that the elements are evenly distributed in the catalyst. Confirmation from TEM image (Fig. 5C) and high magnification TEM image (Fig. 5D) validates that the lattice distance of 0.204 nm corresponds to the (111) plane of metallic cobalt. Additionally, the lattice spacing of 0.34 nm means that the presence of graphene-like structures aids in promoting electron transfer during electrochemical reaction^14–16^. The results mentioned above are consistent with the XRD pattern (Fig. 5E), where the peak at approximately 2θ = 26.3° corresponds to the (002) diffraction peak of graphite and the prominent diffraction peak at around 2θ = 44.2° corresponds to the (111) plane of metallic cobalt (JCPDS 89-4307)^16,17^. It is worth noting that the cobalt metal peaks at 2θ = 51.5°(200) and 75.7°(220) are not obvious, which may be due to the low content of cobalt in the Co-900-50 catalyst, with the cobalt diffraction peaks being overwhelmed by the carbon background peaks^35^. In addition, as can be seen from Fig. 6A, the C(002) diffraction peak becomes increasingly sharper as the temperature increases. This is likely attributable to the enhanced graphitisation degree of the sample with the rising carbonisation temperature, resulting in the distinct formation of the C(002) crystal plane.

As shown in Fig. 6B, the peaks centred at 1350 and 1580 cm^− 1^ in the Raman spectra can be assigned to the D and G bands of carbon materials. Their relative intensity (I_D_/I_G_) is generally employed to estimate the graphitisation degree of the material^36^. The intensity ratio (I_D_/I_G_) of Co-900-50 and Co-900-100 on the Raman spectra is 1.16 and 1.17, respectively, revealing a high degree of graphitisation and guaranteed electrical conductivity of these samples. It indicates Co-900-100 possesses relatively larger defects. Furthermore, an analysis of the specific surface area and pore structure of the prepared samples was conducted using the nitrogen adsorption/desorption technique. Figure 6C illustrates the N_2_ adsorption/desorption curves of the Co-900-50 and Co-900-100 samples, all of which belonged to type IV isotherms. The specific surface areas of the Co-900-50 and Co-900-100 samples are calculated to be 50.3178 m^2^ g^− 1^ and 15.5408 m^2^ g^− 1^. This is consistent with the results of the particle size distribution curve. As seen by the pore size distribution curves in Fig. 6D, there are abundant hierarchical mesoporous structures in both the Co-900-50 and Co-900-100 catalysts. Co-900-50 is primarily composed of microporous and mesoporous structures; However, Co-900-100 contains three pore sizes: microporous, mesoporous, and macroporous. Furthermore, the presence of macropores serves as conduits for gas transport, while mesopores function as the regions for gas-liquid-solid three-phase reactions. The hierarchical porous structure is favourable for electrolyte penetration and oxygen adsorption and desorption during the reaction^37^. Simultaneously, this unique porous structure may also enhance the stability and durability of the catalyst.

**Fig. 7:**
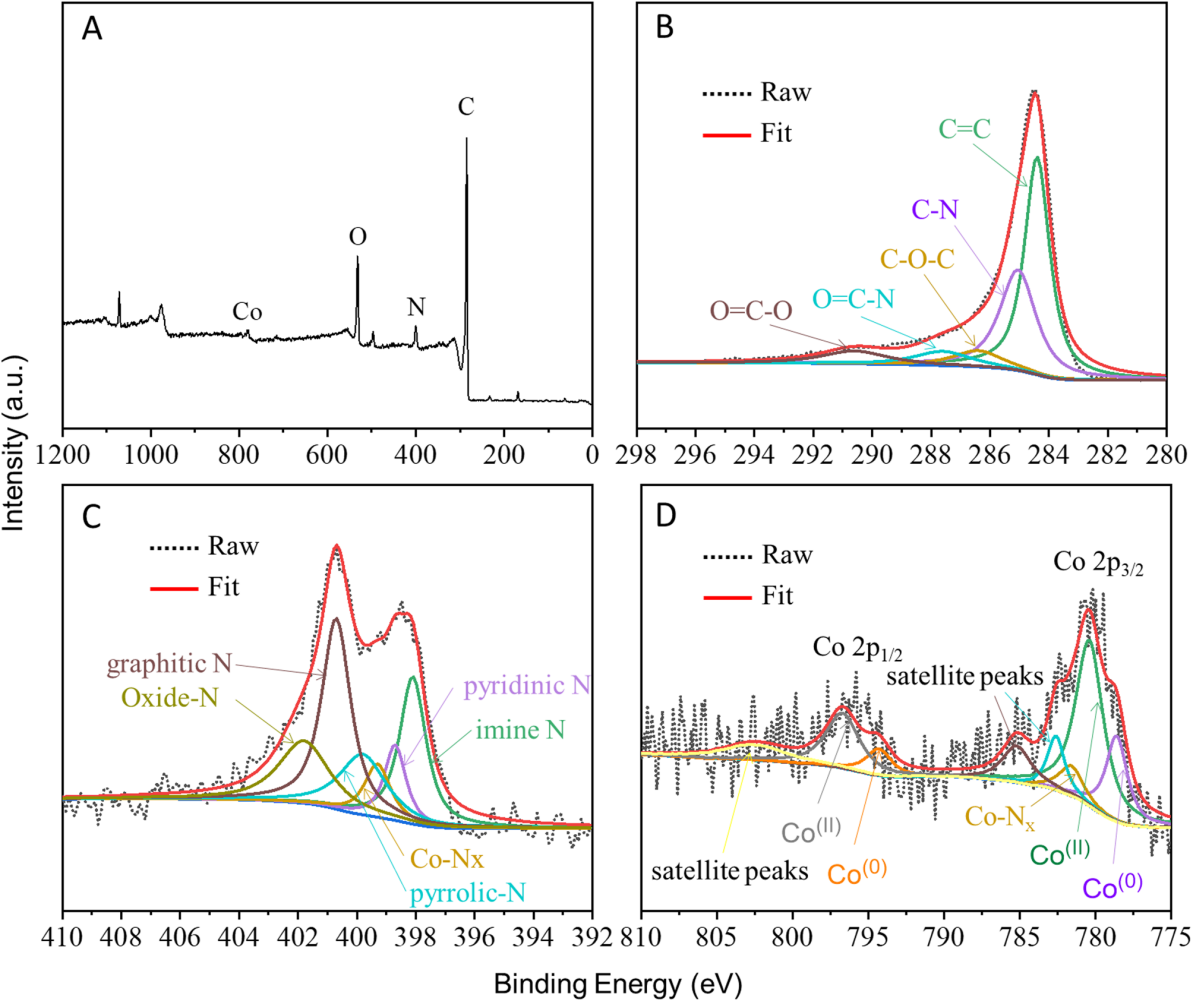
High-resolution XPS spectra, (**A**) survey spectra, (**B**) C 1s, (**C**) N 1s, (**D**) Co 2p.

To further clarify the chemical states of elements in Co-900-50 catalyst, high-resolution XPS spectra are depicted in Fig. 7. The survey spectra (Fig. 7A) indicate the co-existence of carbon (C), cobalt (Co), nitrogen (N) and oxygen (O), which is consistent with the EDX result. The high-resolution XPS spectra of C 1s (Fig. 7B) show a good match between the original data (black dotted line) and the fitting curve (red solid line). The deconvolution peaks in the fitting curve correspond to different groups, namely C=C (284.5 eV), C–N (284.8 eV), C–O−C (286.4 eV), O=C−N (287.6 eV) and O=C−O (290.6 eV)^38,39^. Of these, the strong peak (C = C) is consistent with the (002) diffraction peak of graphite in the XRD pattern, while the peak (C-N) validates the successful incorporation of nitrogen into the lattice of carbon, thereby improving the ORR activity of the active sites^40^. The structures of nitrogen substitutes for carbon atoms can be fitted to several types, as shown in Fig. 7C. The N 1s spectrum reveals six peaks belonging to imine N (398.1 eV), pyridinic N (398.7 eV), Co–N_x_ (399.3 eV), pyrrolic-N (399.8 eV), graphitic N (401.1 eV) and oxidized N (402.2 eV), respectively^41,42^. In the XPS spectra of Co 2p with high resolution (Fig. 7D), according to previous studies, the peaks at about 778.6 eV and 794.3 eV belong to the binding energies of Co^(0)^, as well as the peaks at about 780.4 eV and 796.7 eV belong to the binding energies of Co^(II)^. Additionally, there are the satellite peaks observed at 782.6 eV, 785.2 eV and 802.5 eV. The existence of the Co^(II)^ peak and double satellite peaks at Co 2p_3/2_ typically indicates the existence of Co^(II)^ species in the sample^43,44^. This serves as a crucial characteristic for distinguishing between Co^(II)^ and Co^(III)^ species^45^. In particular, the peak at approximately 781.6 eV indicates the existence of Co–N_x_ species, which is corroborated by the fitting results of high-resolution N 1s XPS spectra.

### Electrochemical properties of the catalysts

**Fig. 8:**
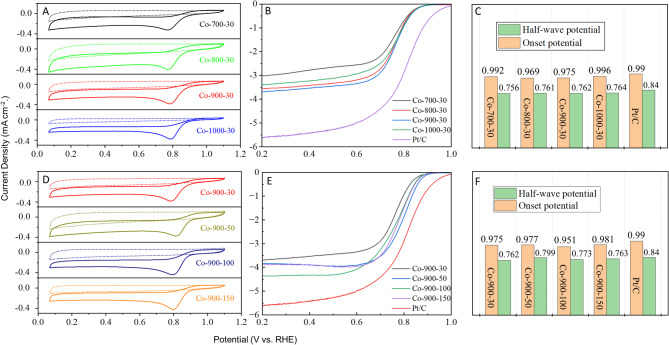
ORR performance of the catalysts in 0.1 M KOH with a sweep rate of 10 mV s^− 1^. (**A**) CV curves, (**B**) LSV curves of the catalysts at 1600 rpm and (**C**) onset potential and half-wave potential (sintering temperature: 700–1000 °C); (**D**) CV curves, (**E**) LSV curves of the catalysts at 1600 rpm and (**F**) onset potential and half-wave potential (the amount of cobalt salt: 30–150 mg).

**Fig. 9:**
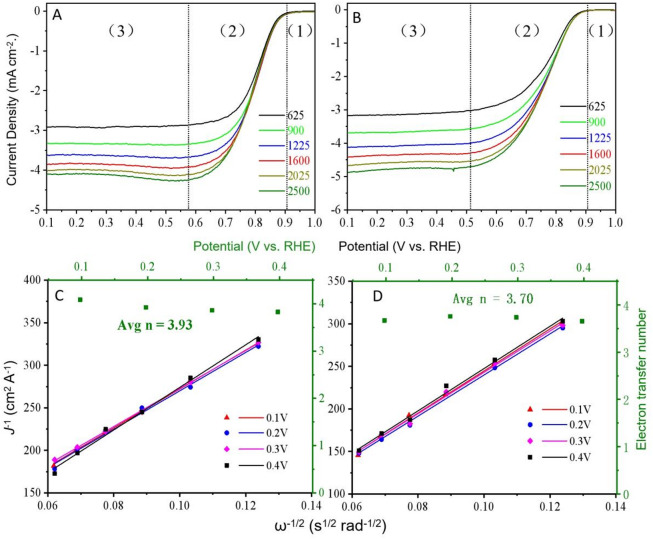
Comparison of ORR performance of the catalysts: ORR polarisation curves of (**A**) Co-900-50 and (**B**) Co-900-100 at different rotating speeds (625 to 2500 rpm), (**C**) The K–L plots of (**C**) Co-900-50 and (**D**) Co-900-100 derived from RDE data and electron transfer number (n) per oxygen molecule at potentials from 0.1 to 0.4 V (vs. RHE).

**Fig. 10:**
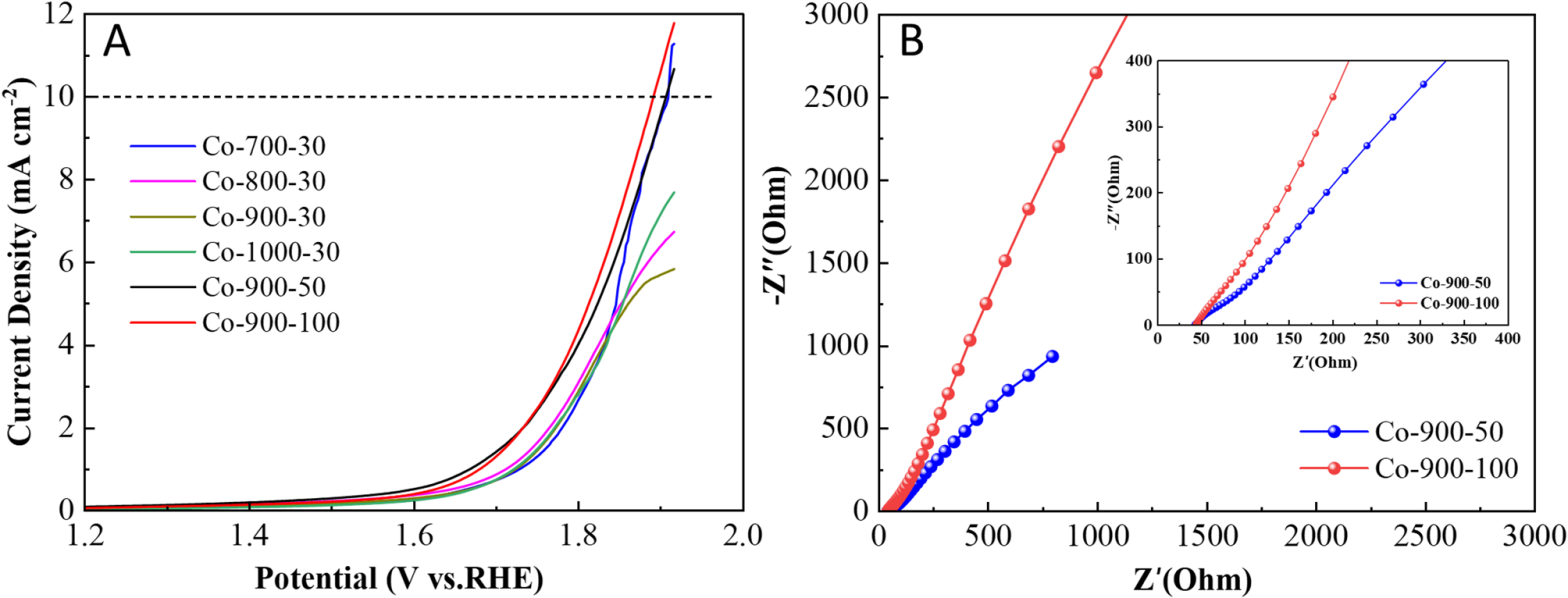
(**A**) OER-LSV curves at 1600 rpm (**B**) Nyquist plots of Co-900-50 and Co-900-100 at open-circuit voltage with a 5mV amplitude and in a frequency range of 0.1–100,000 Hz.

As depicted in Fig. 8A,D, each catalyst displays similar CV curves and the onset potential values for each catalyst are depicted in Fig. 8C,F. These results show that the sintering temperature and the amount of metal salt have little effect on the onset potential of the obtained catalysts in this paper. Conversely, there are clear disparities in the LSV curves for ORR of the catalysts (Fig. 8B,E), and the half-wave potential values for each catalyst are also provided in Fig. 8C,F. The findings reveal that Co-900-50 and Co-900-100 have half-wave potential values of 0.799 and 0.773 V (vs. RHE), respectively. The values are somewhat inferior to those of the commercial 20% Pt/C (0.84 V). Compared to Co-900-50, Co-900-100 demonstrates the highest limiting current density (4.32 mA cm^− 2^), implying that Co-900-100 has better electrical conductivity than Co-900-50. This was verified in the subsequent EIS test results. A detailed comparison of ORR activities of the Co-900-50 and Co-900-100 catalysts with earlier reported catalysts is given in Table S2. Based on Co-900-50 and Co-900-100 exhibiting the highest half-wave potential and limiting current density, the Co-900-50 catalyst and Co-900-100 catalyst are selected for further studies. The polarisation curves (Fig. 9A,B) are obtained at a diverse rotational speed from 625 to 2500 rpm, aiming to explore the ORR kinetics of the catalyst. Intuitively, it includes three regions: (1) kinetic controlled region, (2) mixed kinetic-diffused controlled region and (3) diffused controlled region^46^. In the diffused controlled region, the curves showed no significant deviation in parallelism, suggesting that the reaction against the concentration of dissolved O_2_ followed first-order reaction kinetics. Hence, the K-L plots for Co-900-50 catalyst exhibit significant overlap (Fig. 9C), while the K-L curves for Co-900-100 are almost parallel (Fig. 9D). In line with this, the electron transfer number (n) per oxygen molecule is approximately 3.93 for Co-900-50 and approximately 3.73 for Co-900-100, implying a predominant four-electron ORR pathway. Figure 10A shows the OER-LSV curves and contrast samples collected using an RDE in 0.1 KOH at 1600 rpm. After deducting the theoretical equilibrium potential (1.23 V), the OER overpotential of Co-900-50 and Co-900-100 at 10 mA cm^− 2^ is 0.67 V and 0.66 V, respectively. Both Co-900-50 and Co-900-100 show lower OER overpotentials than Pt/C at 10 mA cm^− 2^. While Co-900-100 displays a better OER activity.

Electrochemical impedance spectroscopy (EIS) measurements were further employed to investigate the catalyst’s electrical conductivity, which is an important factor for electrocatalysis. Ohm resistance (R_s_, Solution Resistance) is the most crucial information in the high-frequency region. It represents the pure resistance generated by the electrolyte between the working electrode and the reference electrode (in a three-electrode system), the electrode materials themselves, and the contact points. This resistance is directly related to the migration of ions and electrons and does not involve Faraday processes. On the Nyquist plot, the intersection point between the high-frequency impedance spectrum and the real axis is typically used to estimate the value of R_s_. As shown in Fig. 10B, the R_s_ of Co-900-50 and Co-900-100 are 43.7 Ω and 42.6 Ω, respectively. The low R_s_ value of Co-900-100 reveals its high electrical conductivity^47^.

Based on the experimental results above, among all samples, Co-900-50 and Co-900-100 exhibit the highest half-wave potential, the lowest overpotential, and relatively high conductivity. They will be selected for assembly into zinc–air batteries to investigate their performance in actual devices.

### Zinc–air battery performance of the catalysts

**Fig. 11:**
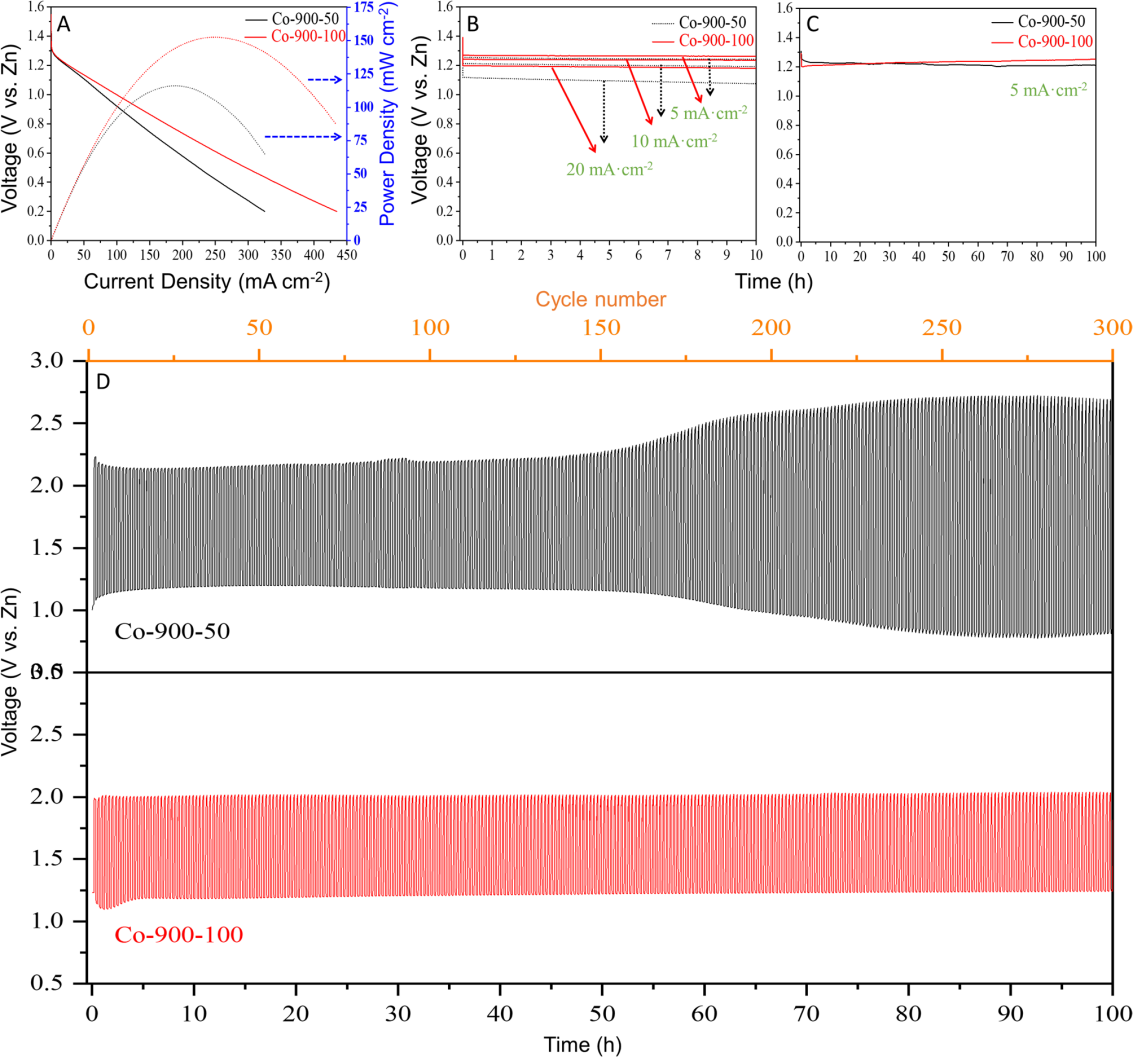
The performances of the zinc–air battery with Co-900-50 catalyst or Co-900-100 catalyst as air cathode. (**A**) Discharge polarization profiles and power density curves of the batteries, (**B**) the long-term discharge tests of the batteries at different current densities, (**C**) the discharge stability of the batteries at 5 mA cm^− 2^ in a total of 100 h, (**D**) a fast charge-discharge cycling test of the battery at current density of 5 mA cm^− 2^ in a total of 300 cycles (100 h).

To further assess the potential of the catalysts, a zinc–air battery (ZAB) device is constructed. The battery consists of a Zn plate serving as the anode, the obtained catalyst served as the cathode catalyst and a mixture solution of 6 M KOH + 0.2 M Zn(Ac)_2_ served as electrolyte. According to the analysis provided, it is anticipated that Co-900-50 and Co-900-100 will exhibit exceptional catalytic efficiency in a zinc–air battery. The discharge polarisation curves (Fig. 11A, black solid line and red solid line) show that the discharge voltage of the Co-900-100 catalyst-based battery is greater than that of Co-900-50. Simultaneously, the peak power density of Co-900-50 catalyst-based battery reaches 116.1 mW cm^− 2^, which is less than that of Co-900-100 (152.5 mW cm^− 2^). The findings indicate that the higher the conductivity of the catalytic material was, the better the actual performance of the zinc–air battery would be. Then, the galvanostatic discharge test is illustrated in the Fig. 11B The discharge platforms of both batteries remain straight without any noticeable voltage drop after 10 h at a current density of 5–20 mA cm^− 2^, indicating that the catalysts possess excellent catalytic efficiency and stability in real-world scenarios. To further assess the practicality of the catalysts, long-term stability tests are performed, and the findings are presented in Fig. 11C. During a discharge process, the battery’s voltage plateau with Co-900-50 catalyst decreases by 0.02 V (from 1.23 to 1.21 V) over a span of 100 h. In contrast, the voltage plateau of the battery with Co-900-100 increases by 0.04 V (from 1.21 to 1.25 V). These findings reinforce the remarkable stability of both the Co-900-50 catalyst and the Co-900-100 catalyst. During the process of electrocatalysis, one of the primary reasons for catalyst deactivation is the corrosion of the carbon support, particularly at elevated potentials. So, galvanostatic charge/discharge cycles can be interpreted as a more demanding test of catalyst stability. As shown in Fig. 11D, it is clearly shown that the Co-900-100 catalyst-based battery exhibits ultra-high stability, where the discharge voltage remains constant at 1.24 V for 300 cycles, each cycle consists of a 10-min discharge instantly followed by a 10-min charge at 5 mA cm^− 2^. Comparatively, the stability of the Co-900-50 catalyst-based battery is slightly inferior to that of Co-900-100, resulting in a decrease in discharge voltage from 1.20 to 0.82 V. Clearly, these findings mean that the zinc–air battery utilising Co-900-100 catalyst shows outstanding cyclic stability owing to the unique structural robustness. A detailed comparison of the zinc–air battery performances with previous studies is given in Table S3.

The summary of the differences in catalyst performance observed in the RDE and zinc air battery is shown in Table S4. Regarding RDE, it is worth noting that Co-900-50 exhibits better ORR performance in terms of half-wave potential and a higher electron transfer number, which is primarily attributable to its large specific surface area^48^. However, when applied in the ZAB, Co-900-50 shows lower ZAB performance than Co-900-100. This is primarily attributable to the following factors: First of all, the testing systems are different. RDE testing is typically conducted in a three-electrode system under ideal conditions (with sufficient O_2_ mass transfer), whereas ZAB testing is a two-electrode system in an air environment, simultaneously constrained by multiple factors, such as catalyst-electrolyte interface stability, mass transfer efficiency, and air electrode integration, rather than being determined solely by the half-cell potential of the ORR^49,50^. Secondly, internal resistance is a key indicator of battery performance. For batteries, the lower the internal resistance, the better the battery performance^51^. Results from the EIS suggest that the higher ZAB performance of Co-900-100 can be attributed to the lower R_s_ (42.6 Ω). Finally, for ZAB, low charge potential (E_charge_), high discharge potential (E_discharge_), and minimal fluctuation of these are required for good rechargeability^52^. In terms of charging, the lower OER overpotential (0.66 V) of the Co-900-100 results in far superior charge voltages (2.0 V). On the other hand, from the discharge polarisation curves (Fig. 11A), Co-900-100 exhibited a higher discharge voltage (1.25 V) compared with Co-900-50 (1.21 V) at 5 mA cm^− 2^. Additionally, a much higher peak power density was observed for Co-900-100 (152.5 mW cm^− 2^) compared with Co-900-50 (116.1 mW cm^− 2^). Furthermore, the ohmic overpotential was a dominant performance loss mechanism in the region between 5 and 450 mA cm^− 2^ for both catalysts based on the observed pseudolinear behaviour from the polarisation curve. In addition to the excellent charge and discharge capabilities, Co-900-100 demonstrates very stable electrochemical durability in terms of both charge and discharge voltages (2.0 V and 1.24 V, respectively) upon galvanostatic cycling up to 300 cycles at 5 mA cm^− 2^ without virtually any voltage losses (Fig. 11D). Based on the above results, the outstanding durability of the Co-900-100 is attributed to unique porous structure, superior battery discharge-charge voltages and significantly smaller ohm resistance.

**Fig. 12:**
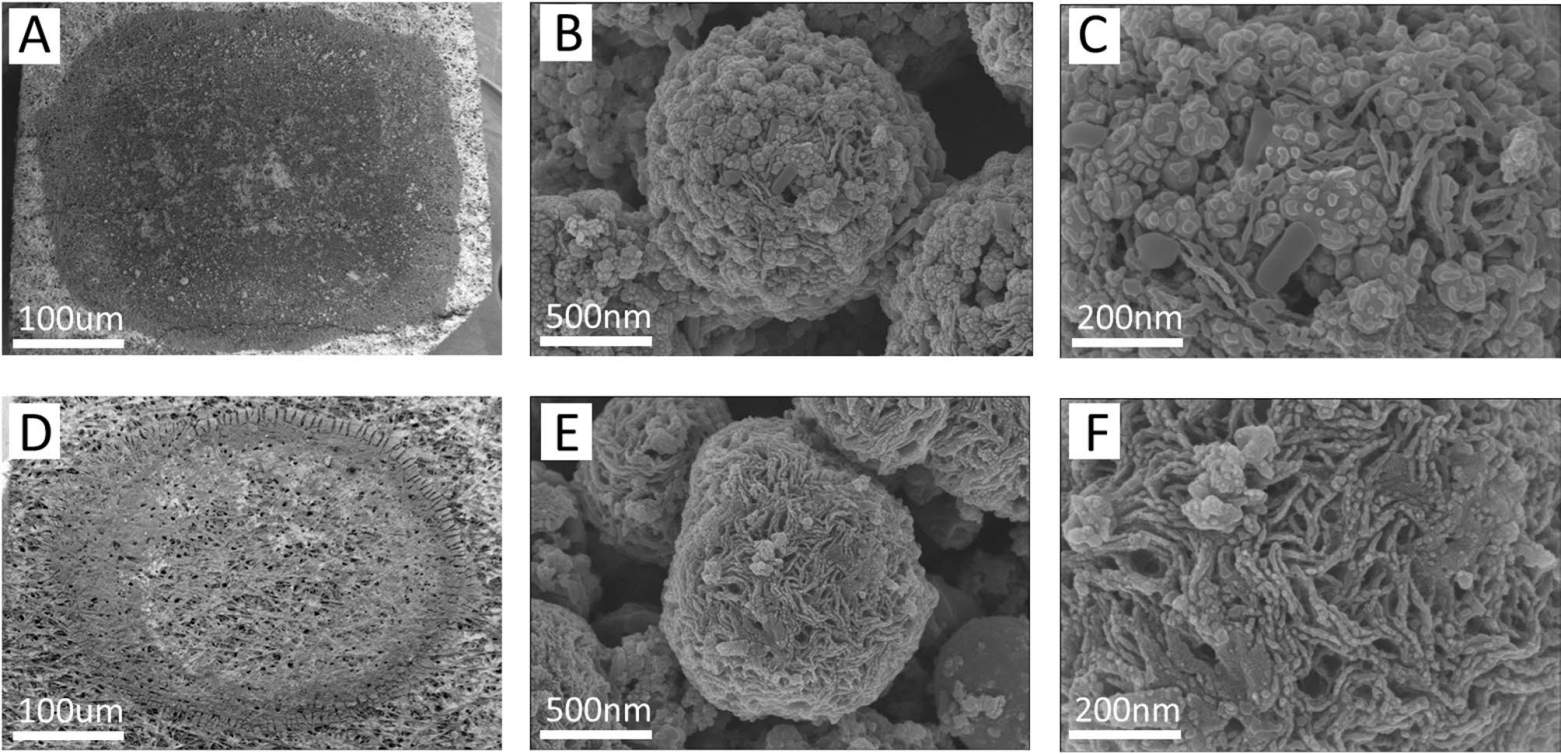
Analysis of the air electrode after discharge–charge 300 cycles at a current density of 5 mA cm^− 2^. (**A**–**C**) SEM image of the Co-900-50 catalyst air electrode, (**D**–**F**) SEM image of the Co-900-100 catalyst air electrode.

**Fig. 13:**
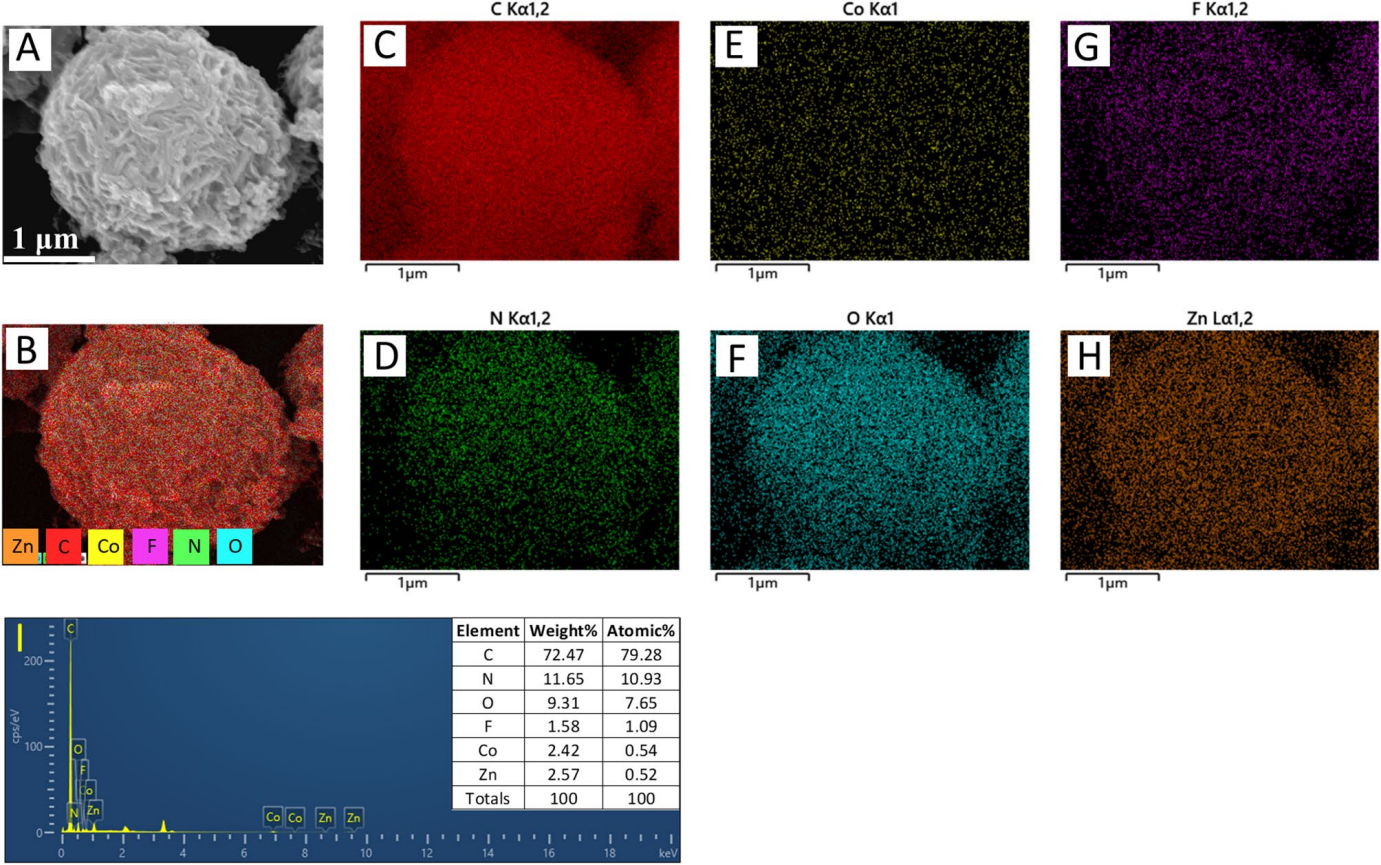
Analysis of the Co-900-50 air electrode after discharge–charge 300 cycles at a current density of 5 mA cm^− 2^. (**A**–**I**) EDX element mapping and spectrum images of Co-900-50 catalyst.

After discharge-charge 300 cycles at a current density of 5 mA cm^− 2^, in order to investigate how the catalyst degrades during excessive cycling, we conducted SEM testing on the cycled catalyst and analysed its morphology. Figure 12 shows the cycled catalyst morphology and structure. It can be clearly seen that the surface of the Co-900-50 catalyst is covered with a large amount of substances (Fig. 12B,C). However, the Co-900-100 catalyst exhibits relatively low surface coverage, likely due to its unique macroporous structure. This characteristic is a key factor contributing to the excellent cycling stability of Co-900-100. Based on the electrochemical reaction of zinc air battery, it is speculated that it may be ZnO. To further determine the material composition of the Co-900-50 catalyst surface after cycling, EDX was conducted, as shown in Fig. 13. The result reveals the presence of the Zn element in the cycled air electrode (Fig. 13H). Additionally, to verify the uniformity of the ZnO coating on the Co-900-50 air electrode surface, EDX mapping was conducted on the constituent elements of the cycled air electrode. Figure 13I shows that the Zn and O elements are observed clearly on the surface of the cycled catalysts, revealing the existence of ZnO on its surface after cycling failure. This is consistent with the findings of previous scholarly research^53,54^. After excessive cycling, the surface of the catalyst is covered with ZnO, which may block the active sites, increase the interfacial impedance, and reduce the electrocatalytic performance of the air electrode, resulting in poor cycling stability of the zinc air battery. This result is consistent with the poor discharge–charge cycling profiles shown in Fig. 11D. As previously mentioned, compared to Co-900-50, Co-900-100 exhibits a three-tiered porous structure comprising micropores, mesopores, and macropores. As demonstrated by SEM images of the cycled catalysts (Fig. 12C,F), the Co-900-50 catalyst with micropores and mesopores is more susceptible to uniform coverage by ZnO during excessive cycling. This leads to blocked active sites, deteriorated cycling stability, and premature failure. In contrast, the macropores within the Co-900-100 catalyst resist ZnO coverage and can store reactants, preserving some active sites and maintaining cycling stability. Consequently, Co-900-100 exhibits the best cycling stability in zinc–air batteries.

The unique, robust nanoporous structure we have constructed exhibits a large specific surface area with abundant pores, including micropores, mesopores, and macropores. It also possesses high electrical conductivity. Notably, the presence of macropores enables the storage of reactants, mesopores enhance reactant transport efficiency, and micropores promote the enrichment of active ions. This achievement fulfils our objective of enhancing the cycling durability of zinc–air batteries while maintaining catalyst efficiency, thereby providing an ideal carrier material for designing high-performance zinc–air batteries.

## Conclusion

In this study, Co–N-doped carbon catalysts were synthesised through the hydrothermal method utilising SiO_2_ as templates. The presence of optimal usage of cobalt salt improves the robustness of the structure, which may be expected to enhance the stability of catalysts. The ORR results validate that Co-900-50 exhibits the greatest half-wave potential, while Co-900-100 demonstrates the highest limiting current density. Furthermore, the performance and durability of the Co-900-50 catalyst and Co-900-100 catalyst are assessed in a zinc–air battery, aiming to enhance their potential for real-world implementation. The findings suggest that the zinc–air battery with the optimal Co-900-100 catalyst showed ultra-high cyclic stability. Specifically, the discharge voltage has remained at 1.24 V throughout 300 charge-discharge cycles. This work provides a valuable approach for synthesising Co–N–C catalysts that exhibit outstanding stability for zinc–air batteries.

## Supplementary Information

Below is the link to the electronic supplementary material.

Supplementary Material 1

## Data Availability

Data will be made available on request. Please contact Fu Niu directly. E-mail Address: jtuniufu123@163.com.

## References

[1] Fu, J. et al. Recent progress in electrically rechargeable zinc–air batteries. *Adv. Mater.* **31**, 1805230 (2019). 10.1002/adma.201805230 30536643

[2] Wang, H., Tang, C. & Zhang, Q. A. review of precious-metal‐free bifunctional oxygen electrocatalysts: Rational design and applications in Zn–air batteries. *Adv. Funct. Mater.* **28**, 1803329 (2018).

[3] Paul, R., Zhu, L., Chen, H., Qu, J. & Dai, L. Recent advances in carbon-based metal‐free electrocatalysts. *Adv. Mater.* **31**, 1806403 (2019). 10.1002/adma.201806403 30785214

[4] Yang, H. et al. Advanced oxygen electrocatalysis in energy conversion and storage. *Adv. Funct. Mater.* **31**, 2007602 (2021).

[5] Kundu, A., Mallick, S., Ghora, S. & Raj, C. R. Advanced oxygen electrocatalyst for air-breathing electrode in Zn–Air batteries. *ACS Appl. Mater. Interfaces*. **13**, 40172–40199 (2021). 34424683 10.1021/acsami.1c08462

[6] Shah, S. S. A. et al. Recent advances on oxygen reduction electrocatalysis: Correlating the characteristic properties of metal organic frameworks and the derived nanomaterials. *Appl. Catal. B: Environ. Energy*. **268**, 118570 (2020).

[7] Yu, P. et al. Co Nanoislands rooted on Co–N–C nanosheets as efficient oxygen electrocatalyst for Zn–air batteries. *Adv. Mater.* **31**, 1901666 (2019). 10.1002/adma.201901666 31169937

[8] He, Y. et al. Atomically dispersed Fe–Co dual metal sites as bifunctional oxygen electrocatalysts for rechargeable and flexible Zn–air batteries. *ACS Catal.* **12**, 1216–1227 (2022).

[9] Thakur, P. et al. Cobalt nanoparticles dispersed nitrogen-doped graphitic nanospheres-based rechargeable high performance zinc–air batteries. *ACS Appl. Energy Mater.* **3**, 7813–7824 (2020).

[10] Zhang, B. et al. Boosting ORR electrocatalytic performance of metal-free mesoporous biomass carbon by synergism of huge specific surface area and ultrahigh pyridinic nitrogen doping. *ACS Sustain. Chem. Eng.* **6**, 13807–13812 (2018).

[11] Wu, M. et al. Ternary doped porous carbon nanofibers with excellent ORR and OER performance for zinc–air batteries. *J. Mater. Chem. A*. **6**, 10918–10925 (2018).

[12] Wei, C. et al. Approaches for measuring the surface areas of metal oxide electrocatalysts for determining their intrinsic electrocatalytic activity. *Chem. Soc. Rev.* **48**, 2518–2534 (2019). 30976760 10.1039/c8cs00848e

[13] Liu, K. et al. Excellent high-rate capability of micron-sized Co-free α-Ni(OH)2 for high-power Ni-MH battery. *J. Alloys Compd.* **768**, 269–276 (2018).

[14] Song, J. et al. A review on fundamentals for designing oxygen evolution electrocatalysts. *Chem. Soc. Rev.* **49**, 2196–2214 (2020). 32133479 10.1039/c9cs00607a

[15] Xiao, C. et al. Co/CoN decorated nitrogen-doped porous carbon derived from melamine sponge as highly active oxygen electrocatalysts for zinc–air batteries. *J. Power Sources*. **453**, 227900 (2020).

[16] Huang, L. B. et al. Engineering carbon-shells of M@NC bifunctional oxygen electrocatalyst towards stable aqueous rechargeable Zn–air batteries. *Chem. Eng. J.* **418**, 129409 (2021).

[17] Yan, L. et al. Formation of sandwiched leaf-like CNTs-Co/ZnCo2O4@NC-CNTs nanohybrids for high-power-density rechargeable Zn–air batteries. *Nano Energy*. **82**, 105710 (2021).

[18] Wu, M., Zhang, G., Wu, M., Prakash, J. & Sun, S. Rational design of multifunctional air electrodes for rechargeable Zn–Air batteries: Recent progress and future perspectives. *Energy Storage Mater.* **21**, 253–286 (2019).

[19] Samanta, A., Ghatak, A., Bhattacharyya, S. & Raj, C. R. Transition metal alloy integrated tubular carbon hybrid nanostructure for bifunctional oxygen electrocatalysis. *Electrochim. Acta*. **348**, 136274 (2020).

[20] Ahmed, S., Shim, J. & Park, G. Co on porous carbon network derived from ZIF-8 for oxygen reduction and evolution reaction in alkaline solution. *Mater. Lett.* **334**, 133727 (2023).

[21] Du, L., Xing, L., Zhang, G. & Sun, S. Metal-organic framework derived carbon materials for electrocatalytic oxygen reactions: Recent progress and future perspectives. *Carbon* **156**, 77–92 (2020).

[22] Duan, X. et al. MOF-derived Co-MOF,O-doped carbon as trifunctional electrocatalysts to enable highly efficient Zn–air batteries and water-splitting. *J. Energy Chem.* **56**, 290–298 (2021).

[23] Cao, Z., Jiang, Z., Li, Y., Huang, C. & Li, Y. Metal–organic gel-derived multimetal oxides as effective electrocatalysts for the oxygen evolution reaction. *ChemSusChem* **12**, 2480–2486 (2019). 30866174 10.1002/cssc.201900194

[24] Guo, Y. et al. 2D hybrid superlattice-based on-chip electrocatalytic microdevice for in situ revealing enhanced catalytic activity. *ACS Nano*. **14**, 1635–1644 (2020). 31994869 10.1021/acsnano.9b06943

[25] Zhang, Z., Sun, J., Wang, F. & Dai, L. Efficient oxygen reduction reaction (ORR) catalysts based on single iron atoms dispersed on a hierarchically structured porous carbon framework. *Angew. Chem.* **130**, 9176–9181 (2018). 10.1002/anie.201804958 29920892

[26] Wang, W. et al. Multi-scale regulation in S, N co-incorporated carbon encapsulated Fe-doped Co9S8 achieving efficient water oxidation with low overpotential. *Nano Res.* **15**, 872–880 (2022).

[27] Wang, W. et al. Vacancy-rich Ni(OH)_2_ drives the electrooxidation of amino C–N bonds to nitrile C≡N bonds. *Angew. Chem.* **132**, 17122–17129 (2020). 10.1002/anie.202005574 32543082

[28] Li, Z. et al. Emerging ultrahigh-density single‐atom catalysts for versatile heterogeneous catalysis applications: redefinition, recent progress, and challenges. *Small Struct.* **3**, 2200041 (2022).

[29] Chakrabarty, S., Mukherjee, A., Su, W. N. & Basu, S. Improved bi-functional ORR and OER catalytic activity of reduced graphene oxide supported ZnCo2O4 microsphere. *Int. J. Hydrog. Energy*. **44**, 1565–1578 (2019).

[30] Lei, X. et al. Fe doped Co9S8 nanoparticles embedded in N, S co-doped porous carbon as an efficient bifunctional electrocatalyst for rechargeable Zn–air batteries. *Electrochim. Acta*. **476**, 143767 (2024).

[31] Zhang, H. et al. Highly dispersed ultrasmall iron phthalocyanine molecule clusters confined by mesopore-rich N-doped hollow carbon nanospheres for efficient oxygen reduction reaction and Zn–air battery. *Chem. Eng. J.* **469**, 143996 (2023).

[32] Lu, T. et al. A versatile extended Stöber approach to monodisperse sub-40 nm carbon nanospheres for stabilizing atomically dispersed FeN sites toward efficient oxygen reduction electrocatalysis. *Small* **2303329**10.1002/smll.202303329 (2023). 10.1002/smll.202303329 37438567

[33] Liu, B. et al. Metal-organic framework assembly derived hierarchically ordered porous carbon for oxygen reduction in both alkaline and acidic media. *Chem. Eng. J.* **430**, 132762 (2022).

[34] Zhang, H. et al. Highly dispersed ultrasmall iron phthalocyanine molecule clusters confined by mesopore-rich N-doped hollow carbon nanospheres for efficient oxygen reduction reaction and Zn–air battery. *Chem. Eng. J.* **469**, 143996 (2023).

[35] Tan, M. et al. Cobalt-nanoparticle impregnated nitrogen-doped porous carbon derived from Schiff-base polymer as excellent bifunctional oxygen electrocatalysts for rechargeable zinc–air batteries. *J. Power Sources*. **490**, 229570 (2021).

[36] Peng, W. et al. ZIF-67-derived Co nanoparticles anchored in N doped hollow carbon nanofibers as bifunctional oxygen electrocatalysts. *Chem. Eng. J.* **407**, 127157 (2021).

[37] Xie, X. et al. Rational construction of FeNi3/N doped carbon nanotubes for high-performance and reversible oxygen catalysis reaction for rechargeable Zn–air battery. *Chem. Eng. J.* **452**, 139253 (2023).

[38] Tu, L., Xiao, Q., Wei, R. & Liu, X. Fabrication and enhanced thermal conductivity of boron nitride and polyarylene ether nitrile hybrids. *Polymers* **11**, 1340 (2019). 31412553 10.3390/polym11081340 PMC6722513

[39] Du, M. et al. Stereoselectively assembled metal–organic framework (MOF) host for catalytic synthesis of carbon hybrids for alkaline-metal‐ion batteries. *Angew Chem. Int. Ed.* **58**, 5307–5311 (2019). 10.1002/anie.201900240 30779319

[40] Zhang, W. et al. ZIF-8/ZIF‐67‐Derived Co‐N_*x*_‐embedded 1D porous carbon nanofibers with graphitic carbon‐encased co nanoparticles as an efficient bifunctional electrocatalyst. *Small* **14**, 1800423 (2018). 10.1002/smll.201800423 29741813

[41] Chen, D. et al. Hierarchical architecture derived from two-dimensional zeolitic imidazolate frameworks as an efficient metal-based bifunctional oxygen electrocatalyst for rechargeable Zn–air batteries. *Electrochim. Acta*. **331**, 135394 (2020).

[42] Chen, S. et al. Uniform virus-like Co–N–Cs electrocatalyst derived from Prussian blue analog for stretchable fiber‐shaped Zn–air batteries. *Adv. Funct. Mater.* **30**, 1908945 (2020).

[43] Lei, X. et al. Fe doped Co9S8 nanoparticles embedded in N, S co-doped porous carbon as an efficient bifunctional electrocatalyst for rechargeable Zn–air batteries. *Electrochim. Acta*. **476**, 143767 (2024).

[44] Feng, X. et al. Revealing the effect of interfacial electron transfer in heterostructured Co_9_ S_8_ @NiFe LDH for enhanced electrocatalytic oxygen evolution. *J. Mater. Chem. A*. **9**, 12244–12254 (2021).

[45] Sun, K. et al. Manipulating the spin state of Co sites in metal–organic frameworks for boosting CO_2_ photoreduction. *J. Am. Chem. Soc.* **146**, 3241–3249 (2024). 38277223 10.1021/jacs.3c11446

[46] Wang, J. et al. Quantitative kinetic analysis on oxygen reduction reaction: A perspective. *Nano Mater. Sci.* **3**, 313–318 (2021).

[47] Cao, Z., Hu, G., Feng, W., Ru, J. & Li, Y. Transport channel engineering between MXene interlayers for Zn–ion hybrid microsupercapacitor with enhanced energy output and cycle stability. *Carbon Neutralization*. **2**, 699–708 (2023).

[48] Xia, J. et al. Fence effect enabling a metal–organic framework-derived single-atom Co–N–C catalyst for high-performance Zn–air batteries. *Langmuir* 4c01976 (2024). 10.1021/acs.langmuir.4c01976 39134089

[49] Yan, L. et al. Atomically precise electrocatalysts for oxygen reduction reaction. *Chem* **9**, 280–342 (2023).

[50] Cai, H. et al. Engineering the local coordination environment and density of FeN_4_ sites by Mn cooperation for electrocatalytic oxygen reduction. *Small* **18**, 2200911 (2022). 10.1002/smll.202200911 35363427

[51] Han, X. et al. A review on the key issues of the lithium ion battery degradation among the whole life cycle. *eTransportation* **1**, 100005 (2019).

[52] Xu, C., Zuo, J., Wang, J. & Chen, Z. Hierarchically structured Mo1–2 C/Co-encased carbon nanotubes with multi-component synergy as bifunctional oxygen electrocatalyst for rechargeable Zn–air battery. *J. Power Sources*. **595**, 234063 (2024).

[53] Sun, W. et al. A rechargeable zinc–air battery based on zinc peroxide chemistry. *Science* **371**, 46–51 (2021). 33384369 10.1126/science.abb9554

[54] Song, Z. et al. Investigation of failure mechanism of rechargeable zinc–air batteries with poly(acrylic acid) alkaline gel electrolyte during discharge–charge cycles at different current densities. *Chem. Eng. J.* **429**, 132331 (2022).

