# Supplementary material for: Synthesis of the porous Co–N-doped carbon catalysts as a durable cathode for zinc–air battery
Source: Sci Rep. 2026 Feb 28;16:11426. doi: 10.1038/s41598-026-40942-4 (PMC13057368; doi:10.1038/s41598-026-40942-4)
Supplement: Supplementary file 1 — Supplementary Material 1 [file 41598_2026_40942_MOESM1_ESM.pdf]

Synthesis of the Porous Co-N-doped Carbon Catalysts as a Durable Cathode for Zinc-air Battery

Fu Niu[1], Jia-Ang Liu[2], Lin-Ting Zhao[3], Yi Qiao[1], Rui-Xia Chu[1], Fang-Yuan Qiu[1], Wan-You Huang[1*]

[1] Key Laboratory of Transportation Industry for Transport Vehicle Detection, Diagnosis and Maintenance Technology, School of Automotive Engineering, Shandong Jiaotong University, Jinan, 250357, China.

[2] Shandong Motor Vehicle Exhaust Pollution Monitoring Centre, Jinan, 250000, China.

[3] Yantai Dongde Industrial Co., Ltd., Yantai, 264006, China.

* Correspondence author.

E-mail: huangwanyou2005@163.com (W. Y. Huang) ;

Table S1: The content of cobalt from Co-900-50 and Co-900-100 was obtained from ICP.

Catalyst	Co (mg/kg)
Co-900-50	20863.68
Co-900-100	35597.16

Table S2：Comparison of ORR electrocatalytic performance of Co-900-50 and Co-900-100 with reported Co-based organic composite catalysts in alkaline solutions.

Catalysts	Onset Potential (V vs. RHE)	Half-wave Potential (V vs. RHE)	Limiting current density（$mA\ cm^{-2}$）	Ref.
Co/N–C	0.93	0.79	4.12	[55]
Co/NHPC-900	0.89	0.78	4.50	[56]
CoNC@NCXS-900	0.94	0.75	4.18	[57]
Co@DUC	0.856	0.72	4.48	[58]
Co-900-50	0.977	0.799	3.91	**This work**
Co-900-100	0.951	0.773	4.32	**This work**
CoN-HPCNF-1000	0.81	0.79	4.49	[59]
Co@N-C700	0.86	0.78	4.48	[60]
MC@NC-0.3	0.990	0.820	4.64	[61]
Co@NGC-NSs	0.920	0.806	4.49	[62]
Pt/C	0.99	0.84	5.52	This work

Table S3： Comparison of zinc-air battery performance with reported Co-based organic composite catalysts in alkaline solutions.

Catalyst	Power density (mW cm^{-2})	Charge/discharge voltage gap (V)	Stability	Ref.
**Co-900-100**	**152.5**	**0.76@5mA cm^{-2}**	**20 min/cycle@5 mA cm^{-2} for 300 cycles, no significant voltage gap change**	**This work**
Co-900-50	116.1	1.1@5 mA cm^{-2}	20 min/cycle@5 mA cm^{-2} for 300 cycles, no significant voltage gap change	This work
Ag/Co/Co3O4 @NC5	120	0.98@10 mA cm^{-2}	20 min/cycle @10 mA cm^{-2} for 160 h, no significant voltage gap change	[63]
Co-N-C-800	89.1	1.42@5 mA cm^{-2}	10 min/cycle @5 mA cm^{-2} for 25 h, no significant voltage gap change	[64]
Co@CNTs	149.2	0.8@2 mA cm^{-2}	20 min/cycle @2 mA cm^{-2} for 120 cycles, no significant voltage gap change	[65]
CoN-HPCNF-900	126.6	0.8@10 mA cm^{-2}	20 min/cycle @10 mA cm^{-2} for 280h, no significant voltage gap change	[66]
Co@NC	141.6	1.07@5 mA cm^{-2}	20 min/cycle @5 mA cm^{-2} for 300 cycles, no significant voltage gap change	[67]

Co/NHPC-800	40	0.99@5 mA cm^{-2}	20 min/cycle @5 mA cm^{-2} for 364 h, no significant voltage gap change	[68]
FeCo/N–C	112.67	1.08@10 mA cm^{-2}	10 min/cycle @10 mA cm^{-2} for 800 cycles, no significant voltage gap change	[69]
Co-SAs/N−C	67.1	1.4@10 mA cm^{-2}	20 min/cycle @10 mA cm-2 for 200 h, no significant voltage gap change	[70]
MC@NC-0.3	153	1.0@10 mA cm^{-2}	20 min/cycle @10 mA cm^{-2} for 300 cycles, no significant voltage gap change	[71]
Co@N-C700	121	0.86@5 mA cm^{-2}	20 min/cycle @5 mA cm^{-2} for 240 h, no significant voltage gap change	[72]

Table S4： Summary of the RDE and ZAB Test Results from Co-900-50 and Co-900-100.

	ORR			OER	EIS	ZAB		
**Catalysts**	Half-wave Potential (V vs. RHE)	Limiting current Density (mA·cm^{-2})	Electron transfer number (n)	Overpotential (V vs. RHE)	R_s (Ω)	Discharge voltage drop (V)	Power density (mW·cm^{-2})	Charge/discharge voltage gap (V)
**Co-900-50**	0.799	3.91	3.93	0.67	43.7	(1.23→1.21,-0.02) 100h@5mA·cm^{-2}	116.1	1.10 20 min/cycle @5 mA·cm^{-2} for 300 cycles
**Co-900-100**	0.773	4.32	3.73	0.66	42.6	(1.21→1.25,+0.04) 100h@5mA·cm^{-2}	152.5	0.76 20 min/cycle @5 mA·cm^{-2} for 300 cycles

Reference

55. Yu, F. *et al.* Enhanced activity towards oxygen electrocatalysis for rechargeable Zn–air batteries by alloying Fe and Co in N-doped carbon. *Dalton Trans.* **50**, 16185–16190 (2021).

56. Zhou, W. *et al.* Co/N-Doped hierarchical porous carbon as an efficient oxygen electrocatalyst for rechargeable Zn–air battery. *RSC Adv.* **11**, 15753–15761 (2021).

57. Jin, H. *et al.* In situ construction of MOF derived CoNC anchored on N-doped carbon xerogel sphere as efficient bifunctional ORR/OER electrocatalyst for Zn-air batteries. *Sci Rep* **15**, 3480 (2025).

58. Fang, W. *et al.* Hierarchical construction of Co nanoparticles embedded in an N doped carbon nanotube/porous nanosheet electrocatalyst for Zn–air batteries. *Catal. Sci. Technol.* **15**, 4291–4302 (2025).

59. Peng, W. *et al.* ZIF-67-derived Co nanoparticles anchored in N doped hollow carbon nanofibers as bifunctional oxygen electrocatalysts. *Chemical Engineering Journal* **407**, 127157 (2021).

60. Li, H., Zhang, M., Zhou, W., Duan, J. & Jin, W. Ultrathin 2D catalysts with N-coordinated single Co atom outside Co cluster for highly efficient Zn-air battery. *Chemical Engineering Journal* **421**, 129719 (2021).

61. Peng, L. *et al.* Efficient MnO and Co nanoparticles coated with N-doped carbon as a bifunctional electrocatalyst for rechargeable Zn-air batteries. *International Journal of Hydrogen Energy* **48**, 19126–19136 (2023).

62. Thakur, P. *et al.* Cobalt Nanoparticles Dispersed Nitrogen-Doped Graphitic Nanospheres-Based Rechargeable High Performance Zinc−Air Batteries. *ACS Appl. Energy Mater.* **3**, 7813–7824 (2020).

63. Wang, T. *et al.* Silver and Polyvalent Cobalt Encapsulated in N-Doped Carbon Nanomaterials as an Efficient Bifunctional Electrocatalyst for Zn-Air Battery. *ChemPhysChem* **26**, e202500496 (2025).

64. Liu, Y. *et al.* Ultra-small cobalt nanoparticles embedded into N-doped hierarchical porous carbon derived from Ion-Exchange MOFs as high-efficient bifunctional catalysts for rechargeable Zn-air battery. *Chemical Engineering Journal* **433**, 134469 (2022).

65. Jiao, J. *et al.* Melamine-assisted pyrolytic synthesis of bifunctional cobalt-based core–shell electrocatalysts for rechargeable zinc–air batteries. *Journal of Energy Chemistry* **53**, 364–371 (2021).

66. Peng, W. *et al.* ZIF-67-derived Co nanoparticles anchored in N doped hollow carbon nanofibers as bifunctional oxygen electrocatalysts. *Chemical Engineering Journal* **407**, 127157 (2021).

67. Tan, M. *et al.* Cobalt-nanoparticle impregnated nitrogen-doped porous carbon derived from Schiff-base polymer as excellent bifunctional oxygen electrocatalysts for rechargeable zinc-air batteries. *Journal of Power Sources* **490**, 229570 (2021).

68. Zhou, W. *et al.* Co/N-Doped hierarchical porous carbon as an efficient oxygen electrocatalyst for rechargeable Zn–air battery. *RSC Adv.* **11**, 15753–15761

(2021).

69. Yu, F. *et al.* Enhanced activity towards oxygen electrocatalysis for rechargeable Zn–air batteries by alloying Fe and Co in N-doped carbon. *Dalton Trans.* **50**, 16185–16190 (2021).

70. Xia, J. *et al.* "Fence" Effect Enabling a Metal–Organic Framework-Derived Single-Atom Co–N–C Catalyst for High-Performance Zn–Air Batteries. *Langmuir* acs.langmuir.4c01976 (2024) doi:10.1021/acs.langmuir.4c01976.

71. Peng, L. *et al.* Efficient MnO and Co nanoparticles coated with N-doped carbon as a bifunctional electrocatalyst for rechargeable Zn-air batteries. *International Journal of Hydrogen Energy* **48**, 19126–19136 (2023).

72. Li, H., Zhang, M., Zhou, W., Duan, J. & Jin, W. Ultrathin 2D catalysts with N-coordinated single Co atom outside Co cluster for highly efficient Zn-air battery. *Chemical Engineering Journal* **421**, 129719 (2021).
